# A scoping review of community health needs and assets assessment: concepts, rationale, tools and uses

**DOI:** 10.1186/s12913-022-08983-3

**Published:** 2023-01-17

**Authors:** Hamid Ravaghi, Ann-Lise Guisset, Samar Elfeky, Naima Nasir, Sedigheh Khani, Elham Ahmadnezhad, Zhaleh Abdi

**Affiliations:** 1 Department of Universal Health Coverage/Health Systems (UHS), World Health Organization, Regional Office for the Eastern Mediterranean, Cairo, Egypt; 2 Department of Integrated Health Services (IHS), World Health Organization, Headquarters, Geneva, Switzerland; 3 Department of Healthier Populations (DHP), World Health Organization, Regional Office of Eastern Mediterranean Region, Cairo, Egypt; 4 Center for Tropical Medicine and Global Health, Nuffield Department of Medicine, University of Oxford, Oxford, UK; 5 National library and Archives of Iran, Tehran, Iran; 6 National Institute of Health Research (NIHR), Tehran University of Medical Sciences, Tehran (TUMS), Tehran, Iran; 7 National Institute of Health Research (NIHR), Tehran University of Medical Sciences, Tehran (TUMS), Tehran, Iran

**Keywords:** Community, Health Needs Assessment, Needs Assessment, Assets Assessment, Population Health, Scoping Review

## Abstract

**Background:**

Community health needs and assets assessment is a means of identifying and describing community health needs and resources, serving as a mechanism to gain the necessary information to make informed choices about community health. The current review of the literature was performed in order to shed more light on concepts, rationale, tools and uses of community health needs and assets assessment.

**Methods:**

We conducted a scoping review of the literature published in English using PubMed, Embase, Scopus, Web of Science, PDQ evidence, NIH database, Cochrane library, CDC library, Trip, and Global Health Library databases until March 2021.

**Results:**

A total of 169 articles including both empirical papers and theoretical and conceptual work were ultimately retained for analysis. Relevant concepts were examined guided by a conceptual framework. The empirical papers were dominantly conducted in the  United States. Qualitative, quantitative and mixed-method approaches were used to collect data on community health needs and assets, with an increasing trend of using mixed-method approaches. Almost half of the included empirical studies used participatory approaches to incorporate community inputs into the process.

**Conclusion:**

Our findings highlight the need for having holistic approaches to assess community’s health needs focusing on physical, mental and social wellbeing, along with considering the broader systems factors and structural challenges to individual and population health. Furthermore, the findings emphasize assessing community health assets as an integral component of the process, beginning foremost with community capabilities and knowledge. There has been a trend toward using mixed-methods approaches to conduct the assessment in recent years that led to the inclusion of the voices of all community members, particularly vulnerable and disadvantaged groups. A notable gap in the existing literature is the lack of long-term or longitudinal–assessment of the community health needs assessment impacts.

**Supplementary Information:**

The online version contains supplementary material available at 10.1186/s12913-022-08983-3.

## Background

The population-based health approach aims to improve the population’s health, promote community resilience and reduce health inequities across the socioeconomic gradient via inter-sectoral partnerships among community groups, government, healthcare systems, and other stakeholders [1]. One key feature for adopting a population-based health approach is to ensure that it is grounded on a solid understanding of community health needs and assets by triangulating evidence from service providers and community members on services availability, accessibility, utilization and experience [2, 3]. The process of identification of unmet health needs in a population is crucial for local authorities seeking to plan appropriate and effective programmes to meet these needs [3, 4]. If these needs are ignored, then there is a risk of a top-down approach for providing health services, reflecting what a few people perceive to be the needs of the population rather than what they actually are [4, 5].

In this context, community health needs assessment is a means of developing a comprehensive understanding of a community’s health and health needs as well as designing interventions to improve community health [6]. Though the process of community health needs assessment can be conducted in several ways, the primary purpose is to provide community leaders or healthcare providers with an overview of local policy, systems, and environmental change strategies currently in place and help to identify areas for improvement [7]. Community health needs assessment can provide them with a more nuanced understanding of the communities they serve, making them aware of pressing issues that require system-level changes and support their efforts for resource mobilization to initiate innovative programmes [8, 9]. The process to gather evidence on community health needs can also serve as a springboard to strengthen community engagement [10].

In general, needs assessments are usually designed to evaluate gaps between current situations and desired outcomes, along with possible solutions to address the gaps. Recently, there has been a trend to move away from framing a community with a deficit perspective (need-based approach) to focus on community assets and resources, called community health needs and assets assessment [11, 12]. In contrast to a need-based perspective which focuses on local deficits and resources outside the community, an asset-based perspective focuses on honing and leveraging existing strengths within the community to address community needs [12–14].

Studies have shown that community health needs assessment is used widely by different users and across different settings [15, 16]. However, these studies varied widely in terms of purpose, process and methods of conducting community health needs assessment. Furthermore, the extent to which an asset-based approach is used is unclear, beyond the inclusion in guidance and recommendations. Thus, to support national or local decision-makers to make informed choices about the scope, tools, methods and use of community health needs and assets assessment, this scoping review of the literature aimed at: 1) Providing conceptual clarity on community health needs and assets assessment, 2) Determining for what purpose and with what methods community health needs and assets assessment are used globally, 3) Drawing the lessons learnt from previous experience with community health needs and assets assessment: what works in what context and under what conditions, 4) Documenting evidence of impact of community health needs and assets assessment, 5) Consolidating tools and methods used to collect evidence/data underpinning community health needs and assets assessment processes.

## Methods

### Search strategy

Ten databases, including PubMed, Embase, Scopus, Web of Science, PDQ evidence, NIH database, Cochrane library, CDC library, Trip, and Global Health Library were searched in February and March 2021. The search strategy was developed through discussion with experts in the field of population health, a research librarian, and a narrative review of the literature. Preliminary search terms were developed by the research team to reflect a number of core concepts including needs, population, needs assessment, assets assessment and participation. The search process was performed by a librarian with expertise in the use of literature databases (SK). The search terms were pilot-tested and agreed upon within the research team. The PubMed database search strategy presented in Additional file 1.

### Inclusion and exclusion criteria

Studies that focus on community health needs and assets assessment in terms of concepts, rationale, uses and tools were considered in both high-income countries (HICs) and low-and middle-income counties (LIMCs). We included studies in the review if they met the following criteria: 1) Papers providing conceptual clarity and explaining rationale for community health needs and (assets) assessment (This can be articles describing community health needs assessment or community assets assessment or community health needs and assets assessments at the same time or separately). The terms capabilities/ strengths/ resources can be used in place of assets and were considered.); 2) Papers describing or evaluating experiences implementing community health needs (and assets) assessment in a single site or multiple sites; 3) Methodological papers describing tools/approaches for community health needs (and assets) assessment; 4) Review of the literature on community health needs (and assets) assessment.

Types of papers not include in the review were: 1) Studies without a clear description of the community health needs and (assets) assessment methods, 2) Studies assessed a single dimension (i.e. health outcomes only, or healthcare providers’ capabilities only such as patient surveys, health outcomes dashboard, health facility assessment), 3) Studies related to a single disease or programme, 4) Studies focused only on engaging individual patient in their own care, and 5) Studies were not in English.

Three reviewers participated in the selection of the relevant studies (HR, ZA, NN). The eligibility and relevance of the articles were determined by two reviewers independently using the above predefined criteria. In the event of disagreement, a consensus was found between all the reviewers about the status of the article.

### Data extraction

Separate data extraction forms were developed for the extraction of the three main categories of papers: conceptual, empirical and review papers. Totally, 121 empirical papers (including 6 review papers) and 48 conceptual and methodological papers were reviewed. Following topics were extracted for empirical papers: 1) General characteristics including author(s), year of publication, country of implementation, study objective(s) and study method; 2) Community health needs and (assets) assessment framing including rational, definitions of community health needs and (assets) assessment/ needs/ assets/ community, initiator(s) or user(s) of the process; 3) Key steps of the process, collected data, data collection tools; 4) Community engagement and the level of engagement; 5) Use of community health needs and (assets) assessment findings, impact of community health needs and (assets) assessment; 6) Facilitators and barriers. Data extraction forms are presented in Additional file 2.

Data extraction forms were pilot-tested prior to the implementation. Two authors (ZA, HR) independently performed a pilot data extraction of a random sample of ten original articles. After piloting, the authors assessed the extracted data in relation to the scoping review questions and revised them accordingly. The content of the form was finalized by discussion within the team. Regarding conceptual papers, two authors (NN and ZA) initially extracted data from three randomly selected papers and subsequently refined and amended the form having research team inputs.

Four reviewers extracted included studies independently. The data extracted were cross-checked by one of the authors and mutual consensus resolved discrepancies. Individual data extraction forms of empirical papers were then merged into a single, unifying document used for the interpretation and presentation of the results. Following typical scoping review methods, the methodological quality of the included articles was not assessed systematically, however, only peer-reviewed articles were included in our review process [17].

### Synthesis of results

**Fig. 1:**
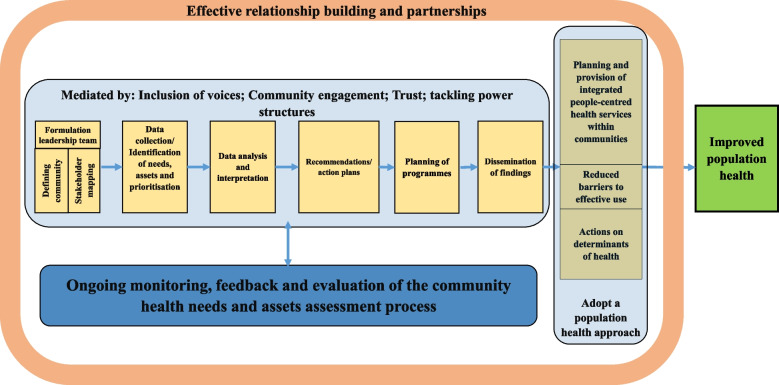
Conceptual framework of the review

Following reading and extracting conceptual papers, a preliminary conceptual framework (Fig. 1) was developed and discussed and agreed upon by team members. The integrative synthesis of the evidence was employed. Specifically, it involved the narrative description of concepts and definitions, key steps of the community health needs assessment and barriers and facilitators of the implementing community health needs assessment.

## Results

**Fig. 2:**
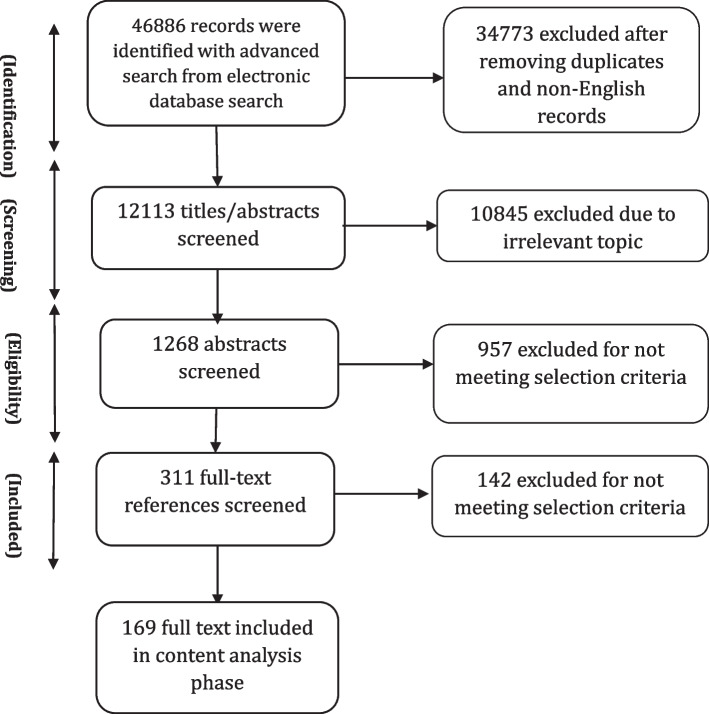
Information flow in scoping review

The study selection process is summarized in Fig. 2. Just over 12,000 records were obtained from the ten databases searched. Articles with obviously irrelevant titles were excluded, as were news items, letters, editorials, book reviews, and articles appearing in newsletters or magazines rather than peer review journals. The remaining abstracts were retrieved, read and assessed. A total of 169 articles including both empirical papers and theoretical and conceptual work were ultimately retained for analysis. A list of all studies with a short description, including the year of publication, key focus, study period, and methods, is presented in Additional files 3 and 4. The first part of the results section focuses on definitions and concepts of community health needs assessment using both conceptual and empirical papers. In the second part of the results section, we describe key steps of the community health needs assessment and tools and methods used to collect data through content analysis of 121 included empirical papers. We also report some important challenges and facilitators faced by included studies while performing community health needs assessment. Role of community participation in the process and the spectrum and types of the participation is discussed in the last part.

### General characteristics of the included studies

The review showed that community health needs assessment is used widely by different users and across different settings in both HICs and LMICs. Among included empirical studies, 81 (out of 121) were conducted in the  United States (US). There were papers from Australia (*n* = 4), South Africa (*n* = 3), Kenya (*n* = 3), Uinted Kingdom (UK) (*n* = 2), Canada (*n* = 2), China (*n* = 2), Dominican Republic (*n* = 2), Republic of Ireland (*n* = 2), Iran (*n* = 2), India (2), Honduras (*n* = 1), Netherland (*n* = 1), Vietnam (*n* = 1), Sudan (*n* = 1), New Zealand (*n* = 1), Madagascar (*n* = 1), Malaysia (*n* = 1), Ecuador (*n* = 1), Indonesia (*n* = 1), Uganda (*n* = 1), Taiwan (*n* = 1), Kyrgyzstan (*n* = 1), Saudi Arabia (*n* = 1), Haiti (*n* = 1), Honduras (*n* = 1) and Korea (*n* = 1).

### Definition of needs

The review showed “need” was a multi-faceted concept with no universal definition. There was a differentiation between “health need” and “healthcare need” in the reviewed literature. Healthcare needs can benefit from health care (health education, disease prevention, diagnosis, treatment, rehabilitation and terminal care). Healthcare providers usually consider needs in terms of healthcare services that they can supply. However, health needs incorporate the wider social and environmental determinants of health, such as deprivation, housing, diet, education and employment. This broader definition allows looking beyond the confines of the medical model based on health services, to the wider influences on health [3].

In this review, relatively few empirical studies focus narrowly on healthcare needs, without attention to other determinants of health that can affect health [18–23]. Most of the included empirical studies looked beyond “physical health needs” to consider wider “social determinants of health” or non-medical factors that can affect a person’s overall health and health outcomes as the conditions—shaped by political, social, and economic forces—in which people are born, grow, live, work, and age [24]. Notably, the need was recognised as a “dynamic concept” whose definition will vary with time according to context and resources available to address these needs [16].

### Definition of community

In general, “community” has been defined as “people with a basis of common interests and network of personal interactions grouped either based on locality or on a specific shared concerns or both” [25]. Shared common interests are particularly important as they can be assessed and, hopefully, met at a community level [26]. Importantly, community is a dynamic concept as individuals can belong to several communities at various times. In our review, community was defined by included studies, particularly those initiated by local authorities or healthcare providers (e.g., hospitals), based on geographical indicators such as county designations or based on the location of the hospital’s/facility’s/authority’s existing or potential service users. Some included empirical studies considered community based on shared interests or characteristics such as race/ethnicity, sexual orientation, or occupation. Medically underserved populations including rural areas [27–30], impoverished urban sectors [31], the homeless [32–35], persons in poverty or of low socioeconomic status, vulnerable children and families [18, 28, 36–38], the elderly [8, 39–42], women and girls [43–47], LGBT (Lesbian, gay, bisexual, and transgender) individuals [48–51], displaced populations, immigrants and racial, ethnic and religious minority groups [12, 19, 36, 42, 52–78] and persons with severe and chronic health problems [79] were considered as a “community” by a number of included studies.

While defining community, a number of its characteristics were determined by included studies including: history, existing groups, physical aspects (i.e. geographic location, community size, its topography and etc.), infrastructure (i.e. health and social care facilities, public transportation, roads, bridges, electricity, mobile telephone services and etc.), demographics (i.e. age, gender, race and ethnicity, marital status, education, number of people in household, first language and etc.), economic conditions, deprivation and/or inequalities, government/politics, community leaders (formal and informal), community culture (formal and informal), existing institutions, crime and community safety, lifestyle and leisure, general health problems and epidemiology.

In our review, community health needs and assets assessment were performed by different organizations as the first step in community health promotion planning, including local health authorities (district/local), community entities [i.e. non-governmental organizations (NGOs), civil society organizations (CSOs), faith-based organizations (FBOs), community-based organizations (CBOs)] and hospitals (public/private). Included studies mostly conducted health needs assessment at the local level (e.g. cities, counties, or other municipalities). The broader understanding of health and its determinants suggests that many public and private entities have a stake in or can affect the community’s health. To engage stakeholders in the process, a number of included empirical studies (*n* = 56, 49%) sought representatives from the community that were best positioned to speak about community health based on their specific knowledge or line of work. These stakeholders were individuals from community and entities who may explicitly be concerned with health or not, which varied by the community context and culture. To have a comprehensive overview of a community needs, it was asserted that defining communities needs to be dynamic and socially constructed to take into account all voices and members, especially those not ordinarily included [80]. Community should be defined in a manner that does not exclude medically underserved, low-income, or minority populations. Integrating community voices is especially important in designing plans and programmes aimed at reducing health disparities in the community [58, 81, 82].

### Definition of assets

Overall, there were limited definitions for “community assets” in the reviewed literature. Assets were described as resources, places, businesses, organizations, and people that can be mobilized to improve the community [11, 83]. This includes members of the community themselves and their capabilities. Assets can therefore be described as the collective resources which individuals and communities have at their disposal, which protect against adverse health outcomes and promote health status [83, 84].

Of 115 included empirical studies, 30 studies addressed community assets while performing community health needs assessment. A wide range of assets, from tangible resources to intangible ones, were considered that can be classified into seven broad categories as follows:

1. Community demographic characteristics: Literacy rates [13], youth population [58, 68], and elderly population [68];
2. Natural capitals: Geographical location and natural resources [21, 81, 85];
3. Economic and financial capitals: Community business [12, 81] community members’ income [21], and housing land ownership [13];
4. Community infrastructure: Level of technology/mobile phone coverage [13, 21], transportation [86], parks and sidewalks [12], sport and recreational facilities [31, 87, 88], public libraries and community centres [88];
5. Community social and educational facilities: Non-profit and non-governmental organizations [59, 87], media [89], educational institutions [12, 31, 81, 90], faith communities [58, 81, 90], and community associations [31];
6. Community health and social facilities: Health and social facilities and providers [72, 81, 85, 86, 89], traditional medicine providers [72], and ongoing health programmes [13, 87];
7. Community’s social and cultural values and resources: Tribal and community culture [58, 68, 74, 91], cultural diversity [81], spirituality and religion [58, 74], strong family bonds and values [59, 74], strong community connections, teamwork and willingness to volunteer [21, 81, 86, 91], mutual support, social support and networks [45, 58, 81, 85], unity, community cohesion and collectivity [21, 59, 74], community capacity [58], community-led activities [86, 91], and community values and traditions [68, 74, 86], resiliency [58], unifying power of communities [13], community administration units e.g. women’s committees [13], an existing group of dedicated healthcare providers [39], a group of concerned citizens [39], community safety [12], the knowledge base of the community members themselves [39] and members’ desire to be healthy [58].

Various qualitative methods such as individual interviews (one-on-one structured conversations) or focus groups (guided, structured, small group discussions) with community members, or key informants’ interviews (formal and informal conversations with leaders and stakeholder groups) or a combination of these methods were reported as the main methods to collect information on community’s assets among reviewed studies. Of these, focus group was the widely used method in community assets assessment [8, 21, 31, 45, 58, 59, 67, 81, 82, 85, 87, 90, 92, 93].

### Definition of community health needs (and assets) assessment

The terms “Community Needs Assessment (CNA)”, “Community Health Needs Assessment (CHNA)”, and “Community Health Needs and Assets Assessment (CHNAA)” were used interchangeably in the literature referring to the process of identifying health needs (and assets) of a given community. Since this review focuses on both community needs and assets, we will use the CHNAA term for the description of the process in this paper.

None of the papers reviewed provided a specific definition for CHNAA. In general, reviewed papers defined CHNAA as: A collaborative, community-engaged, systematic, ongoing, continuous, proactive, comprehensive, cyclical, regular, modifying method or process [28, 33, 69, 92, 94–98]; For the identification, collection, assembly, analysis, distribution, and dissemination of information on key health needs, social needs, concerns, problems, gaps, issues, factors, capabilities, strengths, assets, resources; About communities (or individuals) [21, 23, 28, 31, 33, 37, 41, 45, 54, 79, 89, 94–97, 99–102]; To achieve agreed priorities, create a shared vision, plan actions, garner resources, engage stakeholders, work collaboratively, establish relationships, implement culturally appropriate, multi-sectoral/multilevel intervention strategies, empower residents and enhance community capacity and participation in decision-making process [12, 13, 20, 27, 28, 37, 45, 70, 79, 89, 91, 92, 94, 95, 97–99, 101–104]; Towards improving health and wellbeing, building and transforming health of the communities, increasing community benefits, reducing inequalities; Through which primary/secondary healthcare can respond to local and national priorities [20, 23, 28, 40, 51, 59, 69, 97, 103, 105, 106].

The included studies listed a number of reasons as the rationale for conducting CHNAA. Legislative requirements were most cited as the main rational for conducting CHNAA, particularly among studies conducted in the UK and US. Since the late 1980s, the concept of health needs assessment has gained increasing prominence within the National Health Service (NHS) in the UK. This has been prompted by a series of policy initiatives requiring health facilities to assess needs of their populations and to use these assessments to set priorities to improve the health of their local population [107, 108]. In the US, several national, federal, state, and local funding sources require entities to conduct CHNAA to demonstrate a significant need for their services and programmes to be funded. The most important one is Patient Protection and Affordable Care Act (ACA-2010), requiring non-profit hospitals as tax-exempt entities to perform CHNAAs to maintain non-profit status regularly [92]. Other reasons were mentioned by included studies as the rationales for conducting CHNAA were: lack of information of health needs of a specific community, to facilitate health research and related interventions in a community, to inform the design of contextually relevant programmes and policies, to develop community health improvement plans or health promotion interventions, to develop or update strategic plans, and to receive resources and funds.

### Key steps to conduct CHNAA

The number and nature of CHNAA process steps varied among reviewed studies. However, broadly CHNAAs involved six main steps as follow:

#### Formulation of a leadership team

Forming a leadership team, which was called by different names such as the steering committee/ the research advisory committee (RAC)/ the collaborative task force/ or the community advisory board (CAB), was known as the preliminary step of a CHNAA process. The steering committee was usually composed of local representatives from local agencies and organizations (e.g. non-profit organizations, community service agencies, media outlets, county and municipal governments, colleges and universities, faith-based organizations, and healthcare providers), community members, community stakeholders and leaders, academic partners, health and social officials, and representatives from the investigator body to help guide the development of the CHNAA project.

Leadership team responsibilities were reported as providing inputs on the research purpose, selecting and verifying study methodology and design, providing inputs and feedback on initial survey/topic content and selecting final survey/ topic guide questions, reviewing survey/topic guide length, and ensuring culturally relevant and resonant wording, comprehension and face validity, and monitoring the progress of the data collection. Feedback and recommendations from the steering committee were incorporated throughout the CHNAA process as well. Steering committees usually met on a regular basis.

#### Identification of needs, assets and prioritisation

To collect information on community health, needs and assets, both primary and secondary data were utilized by included studies. Secondary data included information on community socio-demographic and indicators on health status, access, utilization and satisfaction with health and social services at different levels (e.g. community, sub-national and national) to develop a picture of the overall community health. Primary data were collected through quantitative and qualitative methods and mixed-methods approaches.

##### Quantitative studies 

Some empirical studies used individual/household surveys as the only source to identify community needs and concerns (*n* = 28, 24.%). Surveys were a popular method of gathering opinions, preferences and perceptions of needs. Needs assessment surveys typically have written, closed-ended questions filled through the interview (face to face/telephone) or self-completion (paper or online) by community members. Generally, two main kinds of surveys were used by included studies: a) community health assessment survey, and b) community concerns survey. A number of included studies used health assessment surveys as the key data sources of the CHNAA process (*n* = 22, 19%) or along with other types of data, mainly qualitative data (*n* = 21, 18.%). Health assessment surveys typically collected information on demographics, socio-economic variables, respondents’ health status, choice of healthcare providers, and healthcare access issues among community members. Survey questionnaires were mostly developed with inputs from the literature review (similar health assessment surveys conducted at the local or national level), community members and project team discussions. Additional file 5 shows the most important data and indicators collected by included studies through conducting community health  assessment surveys.

Another form of surveys, used alone or in combination with qualitative methods (*n* = 15, 13.5%), was the community concerns survey in which people (community members and/or key informants) are asked to help identify what they see as the most important issues facing their community leading to an inventory of their health priorities [12, 20, 23, 27, 29, 55, 69, 74, 101, 103, 109–113]. A straightforward way to estimate the needs of a community was to simply ask residents their opinion on what particular services are most needed in the community. The focus of this methodology was to create an agenda based on the perceived needs and concerns of community residents. The concerns surveys were based on either focus group discussion with community members and experts or literature review by the researchers or both. Generally, while filling community concerns survey, individuals were asked to rate the importance of each issue in their community on a scale (e.g. 0 = not important, 5 = extremely important) [23, 27, 29, 55, 74, 110]. Participants could also add and rate concerns or service needs that were not listed. Finally, each health problem identified by the community was weighted based on the frequency it was selected on the survey.

General coverage of the surveys was the population aged 18 or over currently residing in the community for a minimum period of time (at least a few months) and able to provide consent for participation. Most surveys were written, closed-ended questions filled through face to face or telephone interviews or self-completion by community members. In addition to the paper-form survey, some studies used email and social media platforms to allow residents to anonymously complete online surveys [29, 51, 57, 96, 103, 110, 114]. A few studies reported that residents received monetary or nonmonetary incentives for their participation upon survey completion [19, 71, 74, 77, 110]. Sampling techniques commonly used are those that promote participation in CHNAAs such as convenience sampling [20, 35, 40, 51, 52, 57, 64, 65, 71, 74, 75, 77, 86, 96, 101, 103, 104, 110, 114, 115]. Only a few studies used random sampling or demonstrated the representativeness of their samples. Their response rates varied between 8 to 95.5%. Most surveys recruited local surveyors and provided them with research training to ensure consistent survey administration to attract community participation. Some studies that assessed health needs among immigrant communities or minority groups recruited bilingual surveyors or/and provided participants with two versions of the instruments, one in the native language to maximize community engagement [12, 27, 52, 65, 71, 86, 103]. Surveys that took a participatory approach to the design, content, terminology, and language level, were reported more understandable and culturally relevant to the community members [52, 65, 75].

Health needs assessment surveys (both concerns surveys and health assessment surveys) reported limitations to data collection based on the assessment timing, data availability, and sample response. As said earlier, using a convenience sampling and non-representative samples, small sample size and inter-rater reliability between surveyors were among some important methodological limitations reported by these studies, which limited the generalisability of the study findings to the entire community population [35, 57, 65, 71, 74, 75, 77, 96, 106, 116]. Convenience sampling method and using community events as sampling sites led to sampling bias in some studies (e.g., an over-representation of some specific groups of the population such as women and low –income or high-income groups) [57, 63, 65, 66, 71, 74, 75, 78, 103, 114, 115].

##### Qualitative studies

Among included studies, about 34% (*n* = 39) used qualitative methods as the main source of data collection on community needs and assets. Some of these studies justified the use of qualitative approach by explaining how the overreliance on quantitative, population-level data resulted in CHNAAs failing to identify health needs and interests of all community members, particularly those of vulnerable population and underrepresented marginalized segments of the community. In addition, these studies concluded that integrating qualitative methods into the CHNAA process has the potential to involve community members in a more participatory fashion, perhaps improving future collaborations between communities and service providers. Such collaborations can help to design focused initiatives, making them more meaningful and culturally appropriate [12, 59, 91, 102].

Key informant interviews, individual interviews with community members, focus groups with community members and community forums were among the qualitative data collection techniques used individually or in combination with each other by these studies to collect data on community needs and assets. They asserted that qualitative techniques specifically targeted to underrepresented segments of the population proved to be effective mechanisms to explore the participants’ perceptions on issues surrounding community health needs and assets. The most used technique to elicit community members’ opinions were focus group discussions and key informant interviews.

Small sample size and single-site setting were mentioned as the most cited limitations of  the qualitative CHNAAs that limit these studies generalisability. Because the studied communities were unique communities with unique assets, constraints, and health needs, the CHNAA findings cannot be generalised to other communities [32, 39, 62, 70, 72, 73, 91, 117, 118]. Another limitation mentioned by some studies was that the demographic composition of the focus group participants, specifically with regards to race, gender, socio-economic status and age group, did not fully reflect the population of studied community as a whole [13, 61, 62, 72, 97, 119]. Some studies reported that they could not include all influencing key informants in the community to facilitate broader understandings of health needs [13, 120].

##### Mixed- methods studies

A variety of data collection methods were used in a number of included studies to ensure that a comprehensive picture of community health needs and resources was obtained (*n* = 48, 42%). Some of these studies were two-phase explanatory mixed-methods studies, with the quantitative phase preceding the qualitative phase (*n* = 14, 12%). They conducted targeted focus groups or community listening sessions or interview with community members/key informants following needs assessment survey to supplement the findings from the survey and provide further information about health status, needs of daily living, barrier to health and access to community resources [8, 21, 41, 53, 55, 66, 67, 93–95, 99, 113, 114, 121]. In addition to these studies, some studies used triangulation mixed-method design to obtain complementary qualitative and quantitative data on community health needs and issues (*n* = 13, 11%). These studies confirmed that using multiple data sources ensured researchers obtain a complete picture of the community health needs. Applying qualitative methods in the form of focus groups and semi-structured interviews enabled exploration of problems and needs within their social context and provided a wider perspective on issues raised. However, to conduct such studies CHNAA teams had to have members who have qualitative and quantitative expertise. There were some limitations specific to the mixed-method studies, including lack of rigor in integrating qualitative and quantitative findings, relying heavily on quantitative data for health need determination, and absence of the voices of the communities most in need [69, 91].

#### Data analysis and interpretation

Qualitative data from focus group discussions and key informant interviews were mainly audio-recorded and transcribed verbatim by the research team and all identifying information was removed. Different analytical approaches, mostly content analysis and thematic analysis, were used to identify main themes related to assets, needs and gaps in the service system and priority populations.

Quantitative data from surveys were analysed using statistical software. Descriptive statistics were used to describe the sample in terms of socioeconomic background and present the prevalence of chronic diseases, risk factors, and health behaviours. Statistical analytical tests were also used to compare results between different groups of community members. Results also were compared by those at the state/ national level or from a similar community. Those diseases or risk factors that had a high prevalence among community members are regarded as priorities that to be addressed further.

#### Formulation of recommendations across various levels (individual, institution, community, policy levels)

Following analysis of the quantitative and qualitative data, the studies included in the review provided a thorough list of health needs and assets of the community. Included studies mainly used CHNAA outputs: 1) as a resource to provide baseline data of community’s health; 2) as a resource to prioritize and plan services; 3) as a resource for writing grant applications; 4) as a resource to guide a comprehensive health promotion strategy.

Not all included CHNAAs proposed interventions to address identified needs and issues. Some of the included studies (*n* = 45, 39%) just provided a snapshot of the most important issues faced by the studied community. They demonstrated several areas where CHNAAs provide more information to researchers, community organizations, and policy-makers. On the other hand, not all identified issues and needs were addressed by those studies performed CHNAA in order to implement interventions or strategies. In practice, specific populations or a number of specific health conditions or health risks, or overarching issues such as health inequality and disparities were prioritized by these studies.

In most cases, decisions on implementation were carried out by the CHNAA steering committees or the research teams. Only a number of studies used a clear and explicit set of criteria for deciding the importance of each issue [22, 27, 43, 67, 94, 118, 122]. A wide range of criteria were used by included studies such as: impact, urgency, community concern, achievability within the set time [94], seriousness, urgency, solvability, and financial burden of the problems [27], perception of survey participants on importance of the identified issues and feasibility of intervention, prevalence, fatality, social and cultural stigma [22], possible interventions, organizational capacity, and community assets and resources [13], importance and possibility of the effecting change [43], prevalence, impact on the duration of sickness, impact on mortality, and the availability of treatment [122], impact of the problem on the overall wellness, quality of life, and resources of their community [118], factors of health issue, size, seriousness, and effectiveness of available interventions [101], importance and feasibility [67].

Different techniques for ranking priorities were applied by included studies such as: 1) Multi-voting technique (decide on priorities by agreeing or disagreeing in group discussions and continuing process/rounds until a final list is developed), 2) Strategy lists (determine if the health needs are of “high or low importance” by placing emphasis on problems whose solutions have maximum impact, with the possibility of limited resource), 3) Nominal group technique (rate health problems from 1 to 10 through group discussion), and 4) Prioritization matrix (weigh and rank multiple criteria for prioritization with numeric values to determine health needs with high importance).

Overall, health priority types were categorized into four main categories by included studies:

1. Medical conditions (e.g. obesity, diabetes, heart diseases, asthma, mental health disorders, substance abuse, vision/ dental problems, HIV/AIDS and sexually transmitted diseases, injuries and health consultations).
2. Health behaviours (e.g. physical activity, eating habits/ nutrition, tobacco consumption, teen pregnancy and violence/gangs).
3. Community conditions (e.g. poverty and unemployment, environmental and infrastructural conditions, such as air quality/pollution, transportation, access to clean water and sanitation, community collaboration, and access to healthy food, exercise facilities and occupational concerns).
4. Health systems priorities (e.g. access to care, including primary care and higher levels of care, specialty care, mental/ behavioural health care and dental care, quality and acceptability of health services, lack of cultural competence in health systems, flexible hours and waiting time).

However, guided by a community-based participatory research (CBPR) approach, a number of studies involved community members and stakeholders in priority identification or ranking [12, 21–23, 27, 29, 31, 36, 41, 43, 49, 53, 55, 56, 58–60, 62, 63, 68, 70, 74, 86–88, 90, 92, 99, 100, 103, 104, 110, 114, 117–119, 121–129], in potential strategy selection [13, 19, 67, 82, 89, 130], and in carrying out strategies [8, 37, 69, 81, 93, 105, 113]. They asserted that by involving the perspectives of the relevant stakeholders, a comprehensive overview of the issues and possible effective solutions was created.

#### Planning of programmes and interventions, implementation and evaluation

The results of CHNAA were used in various ways by included studies. In some studies, particularly researcher-led studies with limited support or involvement of the local authorities, CHNAA just led to the identification of new, locally relevant issues and priorities without any further actions (*n* = 45, 39%). The results of these CHNAAs provided more information to researchers, community organizations, and local policy-makers. Their results also may guide further research agenda in the community [18, 21, 23, 29, 35, 39, 40, 42, 44, 48–50, 52, 54, 55, 62, 64–66, 69–73, 76–78, 85, 96, 106, 122, 123, 131–135]. Some of these studies tried to present their results to the local authorities through various channels in the hope that it would modify existing programmes or implement new ones to meet the needs of the community residents. In addition to identification of relevant issues and priorities, included studies listed at least one outcome associated with the reported CHNAA activity as follows:

1. Development or modification of health and social policy and programmes: The knowledge provided by CHNAAs helped develop better tailored, and thereby potentially more effective interventions by a number of studies. Further, the information gathered from the CHNAA process was used as the baseline against which to measure future targets for assessment efforts and progress in areas were targeted (*n* = 36).
2. Formation of new partnership: In some cases, a new partnership among entities involved in CHNAA was formed to address health issues. One of the partnerships reported successful was the community–academic partnership in which communities used the research capacity of academic institutions to conduct the CHNAAs (*n* = 20). Another type of the partnership reported by some studies was the collaboration among healthcare organizations serving the same geographic area to conduct CHNAA jointly. Conducting a joint CHNAA may avoid duplication of planning efforts and obviate the creation of multiple community health needs assessments for the same population (*n* = 5).
3. Development of new recommendations: Several suggestions were proposed to be considered while designing health improvement interventions in the future by some of the included studies (*n* = 18).
4. Setting or altering strategic direction: Strategic agency direction was established or altered in some cases, which might indicate that the CHNAA was used to redirect resources better to meet the needs of the community (*n* = 4).
5. Raising awareness about health issues: One of the most important insights brought by CHNAA findings was the recognition of the health priorities and contributing factors by the community members, leaders and researchers, leading to an increased awareness of community issues among them (*n* = 8).
6. Engaging and motivating policy-makers and stakeholders: A few studies reported that CHNAAs provided health organizations with the opportunity to identify and interact with key policy-makers, community leaders, and key stakeholders about health priorities and concerns, which might foster a sense of collective ownership and trust in the results and increase the likelihood that the CHNAA will be used (*n* = 5).
7. Having an impact on obtaining resources and resource allocation: The CHNAAs provided the community partners with locally relevant information regarding the current status of health and perceived community needs to inform resource allocation and applications for new grants for the initiation of new programmes (*n* = 14)
8. Contribution to the development of CHNAA process: Some studies reported that the specific methods used in their CHNAA processes could contribute to more relevant and effective community health need assessment process (*n* = 10).

#### Dissemination of findings

Disseminating of the findings and knowledge gained to all partners involved was a foremost step of CHNAAs. The most cited product of the CHNAA process in the included studies was the community needs assessment report. This report includes information about the health of the community as well as the community’s capacity to improve the lives of residents. The report provides the basis for discussion and future actions. In addition to the final report, other channels to disseminate CHNAAs findings were reported as: publishing CHNAA main results in local newspapers, communicating research results with community members and stakeholders in public forums or meetings, presentation results to the steering committee and various stakeholders, posting the report on the local authorities websites, individual meetings with community leaders and stakeholders, posters, and presentation of findings in academic conferences.

### Community participation

Among included studies, around 50 studies (44%) reported using participatory approaches and techniques to encourage community members' participation in CHNAA process. Unlike traditional approaches to health needs assessment, participatory approaches aimed to incorporate community inputs at all stages of the research process to enhance capacity building and overcome barriers to research raised by matters of trust, communication, cultural differences, power and representation. A variety of participatory approaches (e.g. community based participatory research (CBPR), participatory rural appraisal, participatory action research (PAR), rapid participatory appraisal (RPA), tribal participatory research, community-based collaborative action research (CBCAR), precede-proceed model, concept mapping and photovoice) were used by these studies to ensure that communities participate in CHNAA, from defining the community to identifying needs and assets and developing new interventions.

Pennel and colleagues classified the depth of the community participation in CHNAA activities into four main categories [136]. In this classification, depth of the community participation was assessed by the types of activities in which participants were involved throughout the assessment and planning process as follows:

1. No participation: No attempt to engage community stakeholders or members;
2. Consultation-only: Engagement of health-related stakeholders, broader community stakeholders, and/or community members to identify health needs through surveys, interviews, and/or focus groups; verified or validated health needs/priorities with local experts;
3. Moderate participation: Involvement of community stakeholders/ or community members in priority identification; involvement of community stakeholders in strategy selection;
4. Extensive participation: Involvement of community stakeholders/or community members to develop and carry out strategies.

The above classification was used to assess the depth of the community participation by included studies. Based on the content analysis, community participation in CHNAA process varied considerably across the included empirical studies, from minimal to in-depth participation (Table 1). Around 65% of the included studies were involved in consultation-only to identify health needs through one-way communication using tools such as surveys, interviews, and focus group to identify community needs and resources. Around 22% of the included studies solicited moderate participation from the community by involving community in verifying needs and final priority selection and only about 10% of the included studies reported a broad and deep community participation including community involvement in designing and implementing strategies to improve community health.

**Table 1:**
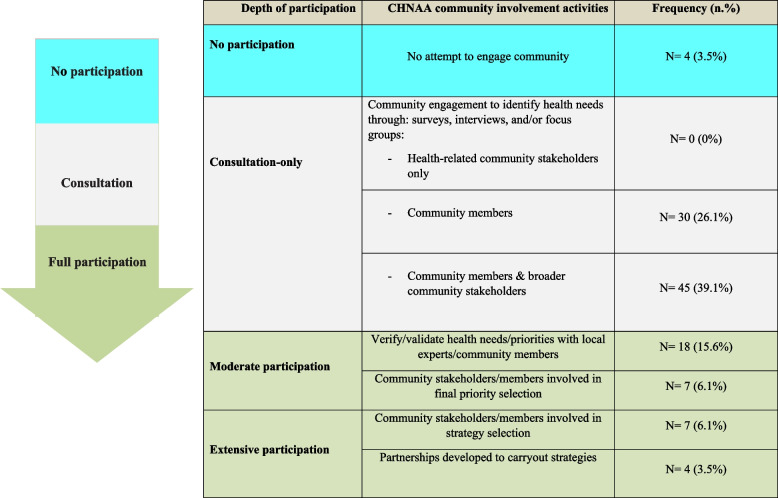
Depth of participation by assessment and planning activity type

### Challenges

Three categories of challenges were cited by the reviewed studies while performing CHNAA projects.

1. Methodological challenges: These are mainly associated with quantitative and qualitative data collection methods, which were discussed earlier. Other methodological challenges cited were: difficulties in aggregating and making sense of data collected from various sources (triangulation), non-generalisability of site-specific data and limitations of the use of existing epidemiological data alone, which does not provide a comprehensive view of health needs, yet is often the most available source of information. Traditional approaches to data collection were challenging where language and literacy barriers existed [12, 52, 65, 71]. Another major challenge reported by studies used community-based participatory research approaches was the challenge of involving the community in decisions related to research design and data collection methods while maintaining an appropriate level of methodological validity and reliability [56, 81, 121]. In addition, participation was not without challenges. Including the perspectives of stakeholders and residents can lead to differing accounts of what services are seen as essential, and each party may push their own agenda based on their personal or professional interests. Further, linguistic and cultural barriers may be a major factor among minority groups hindering participation in such endeavors [81, 137].
2. Logistical challenges: The major logistical challenges reported were the need for a considerable amount of time (often inadequate), and resources required to conduct a comprehensive assessment [80, 138]. Good quality local data on the needs and utilization of health services are usually difficult to obtain [9]. Financial costs are considerable and the depth of information obtained will ultimately depend upon the methods employed [139, 140]. In addition, health professionals, managers and others involved in health services planning and delivery may not have the requisite skills to conduct CHNAAs. This goes beyond technical skills and places an emphasis on soft skills and flexibility including good listening skills, the ability to establish trusting relationships, empathy, working with diverse groups and reflexivity [140, 141]. Moreover, limited health information infrastructure and systems in developing countries settings may have hindered the availability of good quality information to conduct CHNAAs [13, 28, 30, 142].
3. Ethical challenges: Concerns were raised about the ethical issues associated with community consultation about felt needs followed by priority setting process that leaves many needs unaddressed and the bulk of expectations dashed. Labelling, stigma and stereo- typing are other problems raised by needs assessment [143]. Needs assessment results may not be utilised, leaving unmet expectations and may require extensive financial and political support to lead to changes in health service planning and delivery [9]. Comprehensive health needs assessment is likely to produce different, potentially conflicting needs, exposing hidden conflicts and tensions in communities without any mechanisms to address these issues [5]. Further, local participation may only allow those who are able to voice their needs to do so, leaving behind the silent or hidden voices [81]. Involvement of the community in the needs assessment process also impacts upon possible outcomes of the project especially since it is likely that expectations of changes to programmes and service delivery may have arisen from local participation [144].

### Facilitators and enablers

CHNAA projects need to be organized in such a way that they have clear objectives, and are adequately resourced by experienced staff. In addition, factors such as clear objectives, decisive leadership, teamwork, communication, sound study design, adequate resourcing, skilled staff, sufficient time and ownership by stakeholders are among those factors that contribute to the successful implementation of CHNAAs [15, 145]. Most studies cited community participation as a major facilitator of the CHNAA process and outcomes. Participation was shown to foster bidirectional learning and communications, where both health authorities and the community learnt about needs and priorities. Different benefits for community engagement were mentioned by reviewed literature including, improved participants’ recruitment, enhanced capacity among stakeholders, productive conflict resolution, increased quality of outputs and outcomes, increased sustainability of project goals beyond funding and timelines and development of linguistically and culturally appropriate measures. In addition, incorporating community voices has the potential to inform the development of sound measures to tackle health disparities in the basis of race, social class and ethnicity [12, 27, 30, 91, 103, 110, 126, 146].

## Discussion

The main objective of our scoping review was to provide an overview of why and how community health needs and assets assessments (CHNAAs) have been used globally. Substantial variation was found among the studies reviewed concerning definitions, process, participants, methods, goals, and products, yet there were many common characteristics.

Some CHNAAs focused narrowly on health care in assessing needs, with scant attention to other community issues that can affect health. However, most of the included studies looked beyond health needs and considered social and environmental conditions influencing community health. We argue all CHNAAs should approach community health needs assessment holistically, focusing on both individual physical and mental wellbeing as well as casting a social determinants of health lens on the population health.

The review showed that community health needs assessment is used widely by different users and across different settings in both HICs and LMICs. However, in countries such as the US it has become institutionalized and has accordingly been developed, as service providers, particularly hospitals, are mandated to perform CHNAA to compliance with legislative mandates. However, though federal and state laws impose requirements on hospitals to conduct CHNAAs, the methods for needs assessments are generally left to the discretion of each hospital [147]. As a result, assessment methods vary widely. US-based CHNAAs either develop their own CHNAA processes or utilize a process developed at the state or national level to guide their efforts. A number of toolkits have been provided by different organizations across US to help healthcare providers to conduct CHNAA projects [6, 148, 149]. This highlights the need for consensus guidance across many countries and settings while maintaining the responsiveness to contextual needs, assets and priorities.

Both qualitative and quantitative approaches were employed to collect data on community health needs and assets. Overall, there has been a growing use of mixed-methods approaches to conduct CHNAA in recent years, owing to the recognition in the literature that using qualitative and quantitative approaches simultaneously can provide complementary insights determining community health needs and assets [69, 91, 104]. Although quantitative approaches yield concrete evidence of community needs and assets, qualitative approaches provide a context for how these issues can be addressed using available resources [91, 102]. Using qualitative methods in conjunction with more traditional quantitative approaches is especially appropriate for studying complex public health issues and promotes the alignment of implementation plans with the local needs of community members [59, 69, 91]. The growing use of mixed-methods approaches has practical implications for research training and capacity building within entities performing CHNAAs. Organizations who wish to conduct CHNAAs will need to ensure that the competencies and expertise required for mixed-methods studies are available.

Although only a small number of studies provided definitions of assets, there is a growing interest in the literature in asset-based assessment, which examines and mobilizes community assets, instead of focusing on only the needs of communities [11, 84]. Unlike need-based or deficit approaches, asset-based approaches document resources and focus on strengths to enhance and preserve rather than deficits to be remedied. Related to principles of empowerment, it postulates that solutions to community problems already exist within a community’s assets. By recognizing existing capacity, communities can become empowered to take ownership of their health and improve as a population [11, 31, 125]. An asset-based approach was recognized as essential for enhancing trust and community coalitions [83]. Further, it is more participatory in nature through involving community stakeholders throughout the needs assessment process [82, 83]. In particular, it highlights community resilience, resources, and opportunities for positive growth rather than focusing solely on health problems or other concerns [14, 84, 88]. In developing countries, assets identified from within the community are crucial for later use in the implementation of health programmes. The shift from a traditional needs-based perspective to an asset-based perspective to health needs assessment can help to address resource constraints in these countries [13, 30, 150].

There was a growing interest in the use of participatory approaches and in their value in identifying and addressing community health needs over recent years among included studies. About half of the reviewed studies applied CBPR or other community-engaged approaches to perform CHNAA. There are several opportunities to fully engage patients, families, and communities in healthcare delivery redesign to ensure that they are provided in a way that address the community members’ needs and preferences. The CHNAA process is one mechanism for this engagement—and a good precursor to deeper engagement and collaboration [91, 97, 123]. Integrating community voices into CHNAA process may be crucially important for confronting health disparities at the community level, which stemming from socio-historical processes, including racial and ethnic discrimination and economic inequality [33, 74, 86, 91]. To eliminate health disparities, it is critical first to understand social, cultural, and economic determinants of health. CHNAAs, particularly when they include the voices of community residents, can provide an opportunity to understand local processes contributing to health disparities. This knowledge can then be used to inform health and equity initiatives [91, 110, 126]. The development process and implementation of a CHNAA project is an important example of evidence-based public health practice. It is a way to address health and health care disparities experienced by medically underserved populations [86, 92, 126]. Those studies used a participatory approach reported that by having community participation, concerns and issues of the most marginalized and vulnerable populations were voiced. The inclusion of these voices allowed for a broader and deeper understanding of the concerns of those who are typically marginalized and that may be missed in traditional health needs assessment methodologies [33, 56, 58, 74, 86, 110, 137, 146]. Hence, defining communities while performing CHNAA needs to be dynamic and socially constructed to take into account all voices and members especially those not ordinarily included. This deeper understanding is critical to move public health practice and research upstream to address structural and social determinants of health necessary for population-level reductions in health inequities [80, 91].

Although there is widespread theoretical recognition of the importance of in-depth community participation in CHNAA, this has not been fully embraced in practice based on our review. Included studies reported community involvement in various stages of CHNAA with varying depth reflecting a continuum from no participation to extensive participation, in which most studies were located at the middle of the participation continuum. The literature review suggests while certain community stakeholders were engaged in the CHNAA process, most studies did not involve a broad range of stakeholders through adopting a full participation approach. One reason for this could be that for most studies conducted in the US, CHNAA was performed to comply with ACA requirements, which requires hospitals to incorporate inputs of the population served as part of the CHNAA process. Since community inputs as well as the process as a whole is not well-defined by these regulations [20], it seems that the majority of included US-based studies tried to meet legislative requirements by incorporating a minimum level of community and stakeholders’ participation in CHNAA process. In addition, the concept of community engagement in health services planning and implementation has evolved over recent years, from one-way consultative processes to bi-directional collaboration and shared leadership. Although undertaking an in-depth participatory approach through extensive participation of community stakeholders in CHNAAs may pose certain challenges for healthcare providers including requiring additional time and other resources to collaborate with community residents, we argue the benefits to this approach are important to improve health, as reported by some included studies [80, 118, 151].

A notable gap in the existing literature is the lack of long-term or longitudinal–assessment of CHNAA. The review showed that additional research into CHNAA implementation and outcomes is needed. Currently, there are limited data describing the impact of CHNAAs on health outcomes. However, there is ample evidence on different short-term impacts associated with CHNAA implementation, including, the development of health and social interventions, forming the new partnership, raising awareness on health issues, engaging policy-makers, and facilitating obtaining resources. In other words, it is unclear how CHNAA projects are linked directly to health outcomes. Furthermore, the mechanisms between the conduct and use of CHNAA remain largely unknown in the literature [152, 153]. Clearly, not all CHNAA projects result in changes to policies or programmes, and conversely, many programme and policy decisions are made in the absence of CHNAA data [154, 155]. Still, further research to understand these mechanisms and the long term impact of CHNAA is needed to support evidence of its use and value in addressing individual and population health needs.

## Conclusion

This scoping review aimed to provide clarity and supplement the evidence on the key concepts, rationale, methods, tools and outcomes of community health needs and assets assessments (CHNAAs). Importantly, it highlights the need for holistic approaches to needs assessments to focus on physical, mental and social wellbeing, along with considering wider systems factors and structural challenges to individual and population health. Furthermore, the findings emphasize the inclusion of community assets in community health assessments, beginning foremost with community capabilities and knowledge. It is encouraging to see the use of pragmatic approaches including both qualitative and quantitative methods in CHNAA process in the literature. This will help to ensure that a robust and in-depth exploration of needs and assets is available to guide decision making. Although we recognize the challenges with providing consensus on definitions, processes and tools for CHNAA, we argue that more clarity is needed on the key considerations, steps and outcomes for this process across various settings. This study attempts to provide some theoretical insights and empirical information concerning the process, which hopefully will provide useful guidance to community organizations, policy- makers, health service providers and researchers seeking to develop and implement community health needs and assets assessment.

## Supplementary Information

**Additional file 1.** PubMed database search strategy.

**Additional file 2.** Content of the extraction forms.

**Additional file 3.** List of included empirical papers [156–159].

**Additional file 4.** List of included non-empirical papers [160-–175] .

**Additional file 5.** Health indicators collected by community health assessment surveys.

## Data Availability

All data generated or analysed during this study are included in this published article and its supplementary information files.
