# Supplementary material for: A scoping review of community health needs and assets assessment: concepts, rationale, tools and uses
Source: BMC Health Serv Res. 2023 Jan 17;23:44. doi: 10.1186/s12913-022-08983-3 (PMC9847055; doi:10.1186/s12913-022-08983-3)
Supplement: Supplementary file 1 — Additional file 1. PubMed database search strategy. [file 12913_2022_8983_MOESM1_ESM.docx]

**Additional file 1 PubMed database search strategy**

#1. (“Health Services Needs and Demand” [Mesh:NoExp])= 53,915

#2. (((communit* OR populat* OR public) AND ("care need*" OR "treatment need*” OR "healthcare need*" OR "client need*" OR "health need*" OR "service need*" OR "user need*" OR "care priorit*" OR "patient need*" OR "health care demand*" OR "health demand*" OR “unmet need*” OR “health priorit*” OR “healthcare priorit*”)) OR (community need*” OR “population need*” OR “public need*” OR “public demand*” OR “population demand*” OR “community demand*” OR “community health”))[Title/Abstract/Other Term]= 58,093

#3. (1 OR 2) = 106,833

#4. ("Needs Assessment"[Mesh]) = 31,186

#5. (assess* OR addressing OR determin* OR measur* OR identif* OR explor* OR evaluat*) [Title/Other Term] = 2,116,275

#6. (4 OR 5) = 2,141,590

#7. ("Community Networks"[Mesh] OR "Social Capital"[Mesh] OR "Socioeconomic Factors"[Mesh:NoExp] OR "Economic Status"[Mesh] OR "Economic Factors"[Mesh] OR "Social Factors"[Mesh] OR "Social Support"[Mesh:NoExp] OR "Residence Characteristics"[Mesh:NoExp])=261,929

#8. (capabilit* OR capacit* OR "social capital" OR asset* OR resources OR resource OR characteristic*) [Title/Abstract/Other Term] =2,527,021

#9. (7 OR 8) =2,729,687

#10. (3 AND 6 AND 9) =5,545

#11. (Limit: Pub year, 2000-2021) =4,653

#12. (Limit: Exclude; letter to editor, editorial, conference abstracts, comments, book chapters, book reviews) =4,599
