# Supplementary material for: A scoping review of community health needs and assets assessment: concepts, rationale, tools and uses
Source: BMC Health Serv Res. 2023 Jan 17;23:44. doi: 10.1186/s12913-022-08983-3 (PMC9847055; doi:10.1186/s12913-022-08983-3)
Supplement: Supplementary file 2 — Additional file 2. Content of the extraction forms. [file 12913_2022_8983_MOESM2_ESM.docx]

**Additional file 2 Content of the extraction forms**

| 1. **Empirical papers** | | | |
| --- | --- | --- | --- |
| **General characteristics** | Title of the study |  |  |
|  | Author(s) |  |  |
|  | Year of publication |  |  |
|  | Type of manuscript | Options: review of the literature, description of implementation (case study, operational research) |  |
|  | Country/ies of implementation |  |  |
|  | Study objectives (if relevant) |  |  |
|  | Study methods (if relevant) |  |  |
| **Community Health Needs (and Assets) Assessment framing** | Explicit rationale or objectives for implementing CHNAA |  |  |
|  | Is a definition of CHNAA provided? |  |  |
|  | Initiators/target users of CHNAA | Options: district/local authorities, community-based (NGOs/CSOs, healthcare provider), hospital-based (public, private), others |  |
|  | Community definition | Options: local area/district, users/clients, marginalized group, others |  |
|  | Dimensions assessed: needs |  |  |
|  | Dimensions assessed: assets |  |  |
| **Methods for CHNAA** | Key steps in the process of CHNAA implementation |  |  |
|  | Data/indicators collected |  |  |
|  | Data collection tools |  |  |
|  | Gender and equity perspective |  |  |
|  | Stakeholders/community engagement for CHNAA initiation | Whom and how? (were marginalized/vulnerable groups identified?) |  |
|  | Stakeholders/community engagement for data collection | Whom and how? (were marginalized/vulnerable groups identified?) |  |
|  | Stakeholders/community engagement for data analysis, interpretation, and recommendations: | Whom and how? (were marginalized/vulnerable groups identified?) |  |
| **Findings and discussion** | Identified needs |  |  |
|  | Identified assets |  |  |
|  | How has CHNAA been used? |  |  |
|  | What was the impact of CHNAA? How were those documented/assessed? |  |  |
|  | What are lessons learnt from CHNAA (on process)? |  |  |
|  | CHNAA limitations |  |  |
|  | Study limitations |  |  |

| 1. **Conceptual and methodological papers** | |
| --- | --- |
| **General Characteristics** | Title of the study |
|  | Author(s) |
|  | Year of publication |
|  | Type of manuscript |
| Definition of CHNAA |  |
| Definition of community |  |
| Definition of needs |  |
| Definition of assets |  |
| Rationale or purpose of CHNAA |  |
| Methods/tools/frameworks used |  |
| Data collection techniques |  |
| Key steps in the CHNAA process |  |
| Other key elements: gender, equity, empowerment etc. |  |
| Strengths of a framework or method |  |
| Facilitators/ enablers |  |
| Challenges/barriers |  |
| Any other important information |  |
