# Supplementary material for: A scoping review of community health needs and assets assessment: concepts, rationale, tools and uses
Source: BMC Health Serv Res. 2023 Jan 17;23:44. doi: 10.1186/s12913-022-08983-3 (PMC9847055; doi:10.1186/s12913-022-08983-3)
Supplement: Supplementary file 3 — Additional file 3. List of included empirical papers [156–159]. [file 12913_2022_8983_MOESM3_ESM.docx]

**Additional file 3 List of included empirical papers**

| **Ref number** | **Title** | **Author (s)** | **Country** | **Year** | **Method (Qualitative/quantitative/mixed method/review)** |
| --- | --- | --- | --- | --- | --- |
| [121] | The story so far: a mixed-methods evaluation of county-level behavioral health needs, policies, and programs | T. Agrusti et al. | USA | 2020 | Mixed method using participatory approach |
| [27] | Community based needs assessment in an urban area; A participatory action research project | S.S. Ahari et al. | Iran | 2012 | Mixed method using participatory approach |
| [32] | Needs assessment for creating a patient-centered, community-engaged health program for homeless pregnant women | T. Ake et al. | USA | 2018 | Qualitative |
| [110] | Processes and outcomes of a community-based participatory research-driven health needs assessment: a tool for moving health disparity reporting to evidence-based action | T.H. Akintobi et al. | USA | 2018 | Mixed method using participatory approach |
| [94] | Community health needs assessment in Wake County, North Carolina: partnership of public health, hospitals, academia, and other stakeholders | E. Alfano-Sobsey et al. | USA | 2014 | Mixed method |
| [127] | Using CBPR to assess client needs at a social service agency | M.G. Amendola et al. | USA | 2016 | Qualitative using participatory approach |
| [18] | Determinants of child health behaviors in a disadvantaged area from a community perspective: a participatory needs | M. Anselma et al. | Netherlands | 2018 | Qualitative |
| [109] | Applying a basic development needs approach for sustainable and integrated community | M. Asadi-Lari et al. | Iran | 2014 | Quantitative |
| [39] | Voices from the Gila: health care issues for rural elders in south-western New Mexico | J. Averill | USA | 2002 | Qualitative |
| [19] | Identifying health conditions, priorities, and relevant multilevel health promotion intervention strategies in African American churches: a faith community health needs assessment | J. Berkley-Patton et al. | USA | 2018 | Quantitative |
| [52] | Addressing the health needs of high-risk Filipino Americans in the Greater Philadelphia Region | A. Bhimla et al. | USA | 2017 | Quantitative using participatory approach |
| [20] | The impact of community input in community health needs assessments | T.k. Bias et al. | USA | 2017 | Quantitative |
| [29] | Identifying health care needs of rural Ohio citizens: an evaluation of a two-stage methodology | S.W. Birdwell & H. Calesaric | USA | 1996 | Mixed method |
| [53] | Conducting a Hispanic health needs assessment in rural Kansas: building the foundation for community action | M.Bopp et al. | USA | 2012 | Mixed method using participatory approach |
| [79] | A methodological approach to conducting a statewide community needs assessment of pediatric palliative care and hospice resources | K.E. Brock et al. | USA | 2020 | Mixed method |
| [54] | Enhancing adolescent and young adult health services! a review of the community needs assessment process in an urban federally qualified health center | J.C. Burns et al. | USA | 2020 | Qualitative |
| [91] | The power of community voices for enhancing community health needs assessments | C.L. Cain et al. | USA | 2017 | Qualitative |
| [73] | A social needs assessment tool for an urban Latino population | B.A. Careyva et al. | USA | 2017 | Qualitative |
| [47] | Vulnerability multiplied: health needs assessment of 13–18-year-old female orphan and vulnerable children in Kenya | R. Chhabra et al. | kenya | 2018 | Mixed method |
| [21] | Community health needs assessment: a nurses’ global health project in Vietnam | S. Cho et al. | Vietnam | 2018 | Mixed method using participatory approach |
| [81] | Involving communities in community assessment | M.J. Clark et al. | USA | 2003 | Qualitative using participatory approach |
| [51] | The South Carolina LGBT needs assessment: a descriptive overview | J.D. Coleman et al. | USA | 2014 | Quantitative |
| [55] | Richmond Latino needs assessment: a community-university partnership to identify health concerns and service needs for Latino youth | R. Corona et al. | USA | 2009 | Mixed method using participatory approach |
| [15] | The impact of health needs assessment and prioritization on district health board planning in New Zealand | G. Coster et al. | New Zealand | 2009 | Review |
| [156] | The progress of US hospitals in addressing community health needs | G.R. Cramer et al. | USA | 2017 | Review |
| [46] | Women’s knowledge in Madagascar: a health needs assessment study | E.M. Dell et al. | Madagascar | 2012 | Qualitative |
| [117] | An urban American Indian health clinic’s response to a community needs assessment | M.K. Dennis et al. | USA | 2016 | Qualitative using participatory approach |
| [86] | Health equity in community assessments: a participatory approach in rural Virginia | M.J. deValpine & L. H. Trull | USA | 2019 | Mixed method using participatory approach |
| [40] | Health needs assessment of older people in an agricultural plantation | N.C. Din et al. | Malaysia | 2014 | Quantitative |
| [70] | Assessing the health needs of Chinese older adults: findings from a community-based participatory research study in Chicago’s Chinatown | X. Dong et al. | USA | 2010 | Qualitative using participatory approach |
| [56] | Using community-based participatory research to assess health needs among migrant and seasonal farmworkers | E. Doyle et al. | USA | 2006 | Qualitative using participatory approach |
| [8] | A comprehensive, multitiered, targeted community needs assessment model methodology, dissemination, and implementation | D.H. Finifter et al. | USA | 2005 | Mixed method |
| [90] | New medical school engages rural communities to conduct regional health assessment | M. Garrettson et al. | USA | 2010 | Qualitative |
| [57] | A health needs assessment of the Filipino American community in the Greater Las Vegas area | S. Ghimire et al. | USA | 2018 | Quantitative |
| [92] | Community health needs assessment a pathway to the future and a vision for leaders | C. Grant et al. | USA | 2015 | Qualitative |
| [104] | Community health needs assessment for health service planning: realising consumer participation in the health service setting | S. Green et al. | Australia | 2004 | Mixed method using participatory approach |
| [128] | Developing a congregational health needs assessment: lessons learned from using participatory research approach | B. Harmon et al. | USA | 2021 | Quantitative using participatory approach |
| [38] | Child health needs of rural Alabama Latino families | L. Harrison & I Scarinci | USA | 2007 | Qualitative |
| [58] | Enhancing themes and strengths assessment: leveraging academic-led qualitative inquiry in community health assessment to uncover roots of community health inequities | J. Hebert-Beirne et al. | USA | 2017 | Qualitative using participatory approach |
| [59] | Using community-driven, participatory qualitative inquiry to discern nuanced community health needs and assets of Chicago’s La Villita, a Mexican immigrant neighborhood | J. Hebert‑Beirne et al. | USA | 2018 | Qualitative using participatory approach |
| [60] | Oral histories as critical qualitative inquiry in community health assessment | S.G. Hernandez et al. | USA | 2017 | Qualitative using participatory approach |
| [105] | A public health approach to health needs assessment at the interface of primary care and community development: findings from an action research study | M. Horne & J. Costello | UK | 2003 | Mixed methods using participatory approach |
| [89] | Assessing health in an urban neighborhood: community process, data results and implications for practice | M. Idali Torres | USA | 1998 | Quantitative |
| [12] | Using the transformative paradigm to conduct a mixed methods needs assessment of a marginalized community: methodological lessons and implications | K.M. Jackson et al. | USA | 2017 | Mixed method using participatory approach |
| [37] | Responding to rural health needs through community participation: addressing the concerns of children and young adults | V. Jeffery & K. Ervin | Australia | 2011 | Qualitative using participatory approach |
| [74] | Urban Indian voices: a community-based participatory research health and needs assessment | C. Johnson et al. | USA | 2010 | Mixed method using participatory approach |
| [22] | Whose priorities count? Comparison of community-identified health problems and Burden-of-Disease-assessed health priorities in a district in Uganda | L. Kapiriri & O. F. Norheim | Uganda | 2002 | Qualitative using participatory approach |
| [157] | Methodological complexities and the use of GIS in conducting a community needs assessment of a large U.S. municipality | M.J. Kazda et al. | USA | 2009 | Quantitative |
| [35] | Identifying the health service needs of homeless  adults with physical disabilities | M. Kehn & T. Kroll | USA | 2013 | Quantitative |
| [124] | Photovoice in Kenya: using a community-based participatory research method to identify health needs | F.P. Kingery et al. | Kenya | 2016 | Qualitative using participatory approach |
| [33] | Addressing health care needs in the homeless population: a new approach using participatory action research | T. Kiser & L. Hulton | USA | 2018 | Mixed method using participatory approach |
| [99] | A case study of community involvement in a community health needs assessment project in a regional city of Australia | S. Knox & P. Chapman | Australia | 1995 | Mixed method |
| [132] | Collaboration between communities and universities: completion of a community needs assessment | J.C. Kulig & I. Wilde | Canada | 1996 | Qualitative |
| [103] | Development and implementation of a community health survey for public health accreditation: Case study from a rural county in California | K. Kwan et al. | USA | 2018 | Quantitative |
| [123] | Revealing the hidden ‘troubles’ in Northern Ireland: the role of participatory rapid appraisal | A. Lazenbatt et al. | Northern Ireland | 2001 | Mixed method using participatory approach |
| [61] | Assessing the needs and guiding the future: findings from the health needs assessment in 13 Asian American (AA) Communities of Maryland in the United States | S. Lee et al. | USA | 2011 | Qualitative |
| [42] | Health needs of older Aboriginal people in Taiwan: a community based assessment using a multidimensional instrument | L. Lee et al. | Taiwan | 2011 | Quantitative |
| [87] | Rapid appraisal of the health promotion needs of the Hillbrow Community, South Africa | H. Lewis & M.Rudolph | South Africa | 2003 | Qualitative using participatory approach |
| [23] | Community health needs assessment with precede-proceed model: a mixed methods study | Y. Li et al. | China | 2009 | Mixed method using participatory approach |
| [126] | Engaging underserved populations in Affordable Care | A.F. Lightfoot et al. | USA | 2014 | Mixed method using participatory approach |
| [112] | Community health needs, community participation, and evaluation research | J. Louw et al. | South Africa | 1995 | Quantitative |
| [106] | Health needs of a suburban community: a nursing assessment approach | S.P. Lundeen | USA | 1992 | Quantitative |
| [63] | A Community Needs Assessment of Urban Utah American Indians and Alaska Natives | T.R. Mannix et al. | USA | 2018 | Mixed method using participatory approach |
| [62] | Assessing health concerns and barriers in a heterogeneous Latino community | I.L. Martinez & O. Carter-Pokras | USA | 2006 | Qualitative using participatory approach |
| [85] | Exploring community mental health systems – a participatory health needs and assets assessment in the Yamuna Valley, North India | K.R. Mathias et al. | India | 2020 | Mixed method using participatory approach |
| [13] | An asset-focused health needs assessment in a rural community in North India | K.R. Mathias et al. | India | 2015 | Mixed method using participatory approach |
| [131] | Jazan health needs assessment: a key informant approach | W. Milaat et al. | Saudi Arabia | 2007 | Qualitative |
| [76] | Health needs assessment of Plain populations in Lancaster County | K. Miller et al. | USA | 2017 | Quantitative |
| [64] | Assessing the health care needs of Filipino Americans in greater Long Beach | J. Montano et al. | USA | 2009 | Quantitative |
| [31] | Using community-engaged research to explore social determinants of health in a low-resource community in the Dominican Republic: a community health assessment | A. Moore de Peralta et al. | Dominican Republic | 2019 | Mixed method using participatory approach |
| [129] | Collaborative needs assessment and system development in Albama: process and products | B.A. Mulvihill et al. | USA | 1996 | Mixed method |
| [100] | Listening to local voices: adapting rapid appraisal to assess health and social needs in general practice | S.A. Murray et al. | Scotland | 1994 | Mixed method using participatory approach |
| [41] | Health and social service needs of older adults: Implementing a community-based needs assessment | J. Nolin et al. | USA | 2006 | Mixed method |
| [43] | Conducting a needs assessment for women and girls using a gender analysis framework: the Philadelphia Ujima Coalition for a healthier community experience | A.E. Nunez et al. | USA | 2012 | Mixed method using participatory approach |
| [98] | A primary care-based health needs assessment in inner city Dublin | C.M. O’Kelly et al. | Ireland | 2010 | Qualitative |
| [122] | Rapid appraisal of needs in reproductive health care in southern Sudan: qualitative study | C.A. Palmer | Sudan | 1999 | Mixed method using participatory approach |
| [65] | Bangladeshi immigrants in New York City: a community based health needs assessment of a hard to reach population | V.V. Patel et al. | USA | 2012 | Quantitative using participatory approach |
| [78] | A Needs Assessment of Latino men’s health concerns | T. Peak et al. | USA | 2010 | Qualitative |
| [136] | A mixed-methods approach to understanding community participation in community health needs assessments | C.L. Pennel et al. | USA | 2015 | Review |
| [158] | Nonprofit Hospitals’ Approach to Community Health Needs Assessment | C. L. Pennel et al. | USA | 2015 | Review |
| [151] | Community Health Needs Assessment: Potential for Population Health Improvement | C.L. Pennel et al. | USA | 2016 | Review |
| [30] | Understanding community perceptions of health and social needs in a rural Balinese village: results of a rapid participatory appraisal | E. Pepall et al. | Republic of Indonesia (Bali) | 2006 | Qualitative using participatory approach |
| [113] | Community assessment in a vertically integrated health care system | M. Plescia et al. | USA | 2000 | Mixed method |
| [75] | Assessing community health among Indigenous populations in Ecuador with a participatory approach: implications for health reform | B. Puertas & M. Schlesser | Ecuador | 2001 | Quantitative using participatory approach |
| [93] | Leveraging university-community partnerships in rural Georgia: a community health needs assessment template for hospitals | A. Robinson | USA | 2016 | Mixed method using participatory approach |
| [133] | An Innovative model for conducting a participatory community health assessment | A. Running et al. | Honduras | 2007 | Qualitative |
| [125] | The journey to meet emerging community benefit requirements in a rural hospital: a case study | V. Sabin, P. F. Levin | USA | 2015 | Mixed method |
| [114] | A rural local health department–hospital collaborative for a countywide community health assessment | G. Sampson et al. | USA | 2015 | Mixed method |
| [36] | Using community-based participatory research to identify health issues for Cambodian American youth | C. Sangalang et al. | USA | 2020 | Mixed method using participatory approach |
| [130] | Applying community organizing principles to assess health needs in New Haven, Connecticut | A. Santilli et al. | USA | 2016 | Mixed method using participatory approach |
| [71] | Health assessment of the Arab American community in Southwest Brooklyn | L. Sarsour et al. | USA | 2010 | Quantitative |
| [34] | Service learning: a strategy for conducting a health needs assessment of the homeless | M. Schaffer et al. | USA | 2000 | Quantitative |
| [118] | Community health needs assessment in a rural setting: foundation for a community–academic partnership | D. L. Schutte et al. | USA | 2016 | Mixed method using participatory approach |
| [28] | Community health needs assessment for a child health promotion program in Kyrgyzstan | H. Shin et al. | Kyrgyzstan | 2018 | Mixed method |
| [134] | Adapting developing country epidemiological assessment techniques to improve the quality of health needs assessments in developed countries | S.M. Smith et al. | Ireland | 2004 | Quantitative |
| [153] | Reviewing self-reported impacts of community health assessment in local health jurisdictions | C. Spice & K. Snyder | USA | 2009 | Review |
| [101] | Health Watch 2000: community health assessment in South Central Ohio | S.A.R. Stanley & S. Stein | USA | 1998 | Quantitative |
| [48] | health needs and experiences of a LGBT population in Georgia and South Carolina | L.M. Stepleman et al. | USA | 2018 | Quantitative |
| [66] | Assessing health needs in African American churches: a mixed‑methods study | D. Su et al. | USA | 2019 | Mixed method |
| [67] | Community health needs assessment and action planning in seven Dominican Bateyes | S.V. Suiter | Dominican Republic | 2016 | Mixed method using participatory approach |
| [88] | Critiquing Indicators of Community Strengths in Community Health Needs Assessments | C. Talmage | USA | 2021 | Mixed method using participatory approach |
| [77] | Investigating the myth of the ‘‘model minority’’: a participatory community health assessment of Chinese and Vietnamese adults | S.A. Tendulkar et al. | USA | 2012 | Mixed method using participatory approach |
| [72] | Health needs in Brooklyn’s Chinatown: a pilot assessment using rapid participatory appraisal | K. Thein et al. | USA | 2009 | Qualitative using participatory approach |
| [68] | Identifying community needs and resources in a native community: a research partnership in the Pacific Northwest | L.R. Thomas et al. | USA | 2010 | Qualitative |
| [120] | A Community mental health needs assessment of a racially and ethnically diverse population in New England: narratives from community stakeholders | R.A. Torres Stone et al. | USA | 2019 | Qualitative |
| [44] | Unmet health needs identified by Haitian women as priorities for attention: a qualitative study | R.P. Urrutia | USA | 2012 | Qualitative |
| [82] | Trialing the community-based collaborative action research framework: supporting rural health through a community health needs assessment | S.A. Van Gelderen et al. | USA | 2018 | Mixed method using participatory approach |
| [116] | The unmet needs and health priorities of the urban poor: generating the evidence base for urban community health worker programmes in South Africa | A. van Rie et al. | South Africa | 2018 | Quantitative |
| [159] | Identifying the health needs in rural Appalachian Ohio: outcomes of a rural community-academic partnership | R.A. Vance and et al | USA | 2008 | Mixed method |
| [119] | “One program that could improve health in this neighborhood is ?” using concept mapping to engage communities as part of a health and human services needs assessment. | A. J. Velonis et al. | Canada | 2018 | Qualitative |
| [115] | Addressing a Growing Community’s Health Needs: Project SAHNA (South Asian Health Needs Assessment) | A.Vyas et al. | USA | 2013 | Quantitative |
| [95] | ‘Don’t fix what ain’t broke’: evaluating the effectiveness of a Men’s Shed in inner-regional Australia | A. Waling & D. Fildes | Australia | 2016 | Mixed method |
| [69] | Using a mixed-methods approach to identify health concerns in an African American community | B. Weathers et al. | USA | 2011 | Mixed method using participatory approach |
| [49] | The sexual health needs of sexual minority women in Western Kenya: An exploratory community assessment and public policy analysis | B. D. M. Wilson et al. | Kenya | 2019 | Qualitative |
| [96] | An online, quantitative community health needs assessment of Hale‘iwa and Waialua, O‘ahu, Hawai‘i | N. A.K. Witten et al. | USA | 2020 | Quantitative |
| [135] | Community-based health needs assessment in Léogâne and Gressier, Haiti: six years post-earthquake | E.A. Wood et al. | Haiti | 2017 | Qualitative |
| [97] | A community health needs assessment using principles of community based participatory research in a Mississippi Delta community: a novel methodological approach | C.D. Woodyard et al. | USA | 2012 | Mixed method using participatory approach |
| [102] | Assessment of community health needs of Chongqing residents: a qualitative study | L.Ying et al. | China | 2008 | Qualitative |
| [111] | Community health service needs assessment in Korea using OMAHA Classification System | I.Y. Yoo et al. | Korea | 2004 | Quantitative |
| [50] | Empowerment through accessibility: community needs assessment data for LGBTQ communities | C. Zajac & K. C. Godshall | USA | 2020 | Qualitative |
| [45] | Application of a gender-based approach to conducting a community health assessment for rural women in Southern Illinois | K. Zimmermann et al. | USA | 2014 | Mixed method |
