# Supplementary material for: A scoping review of community health needs and assets assessment: concepts, rationale, tools and uses
Source: BMC Health Serv Res. 2023 Jan 17;23:44. doi: 10.1186/s12913-022-08983-3 (PMC9847055; doi:10.1186/s12913-022-08983-3)
Supplement: Supplementary file 4 — Additional file 4. List of included non-empirical papers [160-–175] . [file 12913_2022_8983_MOESM4_ESM.docx]

**Additional file 4 List of included non-empirical papers (conceptual/methodological papers)**

| **Ref number** | **Title** | **Author (s)** | **Year** | **Conceptual/methodological** |
| --- | --- | --- | --- | --- |
| [139] | Community health needs assessments: filling data gaps for population health research and management | P. Alberti | 2014 | Methodological |
| [11] | Needs assessment and asset/capacity building: a promising development in practice | J. W. Altschuld et al. | 2014 | Methodological |
| [149] | Conducting community health needs assessments in rural communities: lessons learned | K. Becker | 2015 | Methodological |
| [160] | The importance of individual-site and system-wide community health needs assessments | T. Bias et al. | 2020 | Conceptual |
| [161] | Approaches to community needs assessment: a literature review | J. Billings & S. Cowley | 1994 | Conceptual |
| [162] | Assessing health service needs: tools for health planning | K. Brazil & M. Anderson | 1996 | Methodological |
| [163] | Assessing community healthcare needs: lessons from Africa | S. Campbell | 2015 | Conceptual |
| [164] | Joint community health needs assessments as a path for coordinating community-wide health improvement efforts between hospitals and local health departments | E. L. Carlton & S.R. Singh | 2018 | Methodological |
| [147] | Tax-exempt hospitals, community health needs and addressing disparities | M. Crossly | 2012 | Methodological |
| [145] | Evaluation of community health assessment in Kansas | D. Curtis | 2002 | Methodological |
| [165] | Population-based needs assessment bringing public health to midwifery practice | E. R. Declercq et al. | 1997 | Methodological |
| [154] | Funding priorities: data-driven approach for prioritizing community health needs in vulnerable communities | H J-M. Diaz et al. | 2019 | Methodological |
| [166] | Community health needs assessments: expanding the boundaries of nursing education in population health | R. Evans-Agnew et al. | 2016 | Methodological |
| [80] | Defining “community” in community health evaluation: perspectives from a sample of nonprofit Appalachian hospitals | B. Franz et al. | 2017 | Conceptual |
| [10] | Is community health assessment worthwhile? | D. Friedman & R. Gibson Parrish | 2009 | Conceptual |
| [2] | Use of community health needs assessment for regional planning in country South Australia | J. Fuller | 2001 | Methodological |
| [83] | “Accentuate the positive!”: using an asset mapping tool as part of a community-health needs assessment | K. Goldman& K J. Schmalz | 2005 | Conceptual |
| [167] | Participation, health and the development of community resources in Southern Brazil | P.A. Guareschi & S. Jovchelovitch | 2004 | Conceptual |
| [143] | Needs assessment must become more change-focused | P. Hawe | 1996 | Conceptual |
| [108] | The influence of health needs assessment on health care decision-making in London health authorities | M. Hensher & N.Fulop | 1999 | Methodological |
| [168] | A community assessment model appropriate for the Iranian community | K. Holakouie et al. | 2014 | Methodological |
| [138] | Community health assessment: driving the need for current, easily accessible population health data | P. Irani et al. | 2006 | Methodological |
| [169] | Review of community-based research: assessing partnership approaches to improve public health | B.A. Israel et al. | 1998 | Methodological |
| [26] | Community profiling as part of a health needs assessment | K. Jack & M. Holt | 2007 | Conceptual |
| [107] | Making sense of health needs assessment | J. Jordan & J. Wright | 1997 | Conceptual |
| [5] | Health needs assessment, whose priorities? Listening to users and the public | J. Jordan et al. | 1998 | Conceptual |
| [146] | Using community-partnered participatory research to address health disparities in a Latino community | S. Kim et al. | 2005 | Methodological |
| [170] | Community Health Needs Assessments: A Framework for America’s Hospitals | C. J. King, & J. L. Roach | 2015 | Methodological |
| [14] | Putting the community back into community health needs assessments: maximizing partnerships via community-based participatory research | C. M. Kirk et al. | 2017 | Methodological |
| [137] | Conducting an assessment of health needs and resources in a racial/ethnic minority community | M. Lillie-Blanton & S. C. Hoffman | 1995 | Methodological |
| [171] | Community needs and strengths assessments as an active learning project | R. Misra & D.Ballard | 2003 | Methodological |
| [6] | Criteria for assessing the usefulness of community health assessments a literature review | S. Myers & M. A. Stoto | 2006 | Methodological |
| [16] | Needs-led assessment: the challenges and the reality | B. Parry-Jones & J. Soulsby | 2001 | Conceptual |
| [150] | Guidelines for conducting rapid participatory appraisals of community health needs in developing countries: experience from Tulikup, Bali | E. Pepall et al. | 2015 | Methodological |
| [172] | A Content analysis of nonprofit hospital community health needs assessments and community benefit implementation strategies in Philadelphia | R.E. Powell et al. | 2017 | Methodological |
| [173] | Community analysis for health planning: strategies for primary care practitioners | S.M. Reece | 1993 | Methodological |
| [34] | Assessing community health needs in a rural area: determining best practices to meet new Affordable Care Act requirements | E. Schafer & R.S. Dawson | 2015 | Methodological |
| [148] | A review of tools to assist hospitals in meeting community health assessment and implementation strategy requirements | K.E. Schifferdecker et al. | 2016 | Methodological |
| [84] | Assets-Oriented Community Assessment | P.A. Sharpe et al. | 2000 | Conceptual |
| [140] | What challenges do nonprofit hospitals face in taking on community health needs assessments? a qualitative study from Appalachian Ohio | D. Skinner et al. | 2017 | Methodological |
| [142] | Community health assessment opportunities and challenges in the 21st century: implications for professional development | L.U. Smith et al. | 2017 | Methodological |
| [152] | Effective community health assessments in King County, Washington | D. Solet et al. | 2009 | Methodological |
| [9] | Needs assessment: from theory to practice | A. Stevens & S. Gillam | 1991 | Conceptual |
| [174] | A web-based tool for assessing and improving the usefulness of community health assessments | M.A. Stoto et al. | 2009 | Methodological |
| [141] | Making better use of population health data for community health needs assessments | M.A. Stoto et al. | 2019 | Methodological |
| [175] | Voices across Kansas: community health assessment and improvement efforts among local health departments | R.E. Wetta et al. | 2014 | Methodological |
| [4] | Assessing health needs in developing countries | J. Wright & J. Walley | 1998 | Conceptual |
| [3] | Development and importance of health needs assessment | J. Wright et al. | 1998 | Conceptual |
