# Supplementary material for: A scoping review of community health needs and assets assessment: concepts, rationale, tools and uses
Source: BMC Health Serv Res. 2023 Jan 17;23:44. doi: 10.1186/s12913-022-08983-3 (PMC9847055; doi:10.1186/s12913-022-08983-3)
Supplement: Supplementary file 5 — Additional file 5. Health indicators collected by community health assessment surveys. [file 12913_2022_8983_MOESM5_ESM.docx]

| **Additional file 5 Most frequently used health indicators by community health assessment surveys** | | | | | |
| --- | --- | --- | --- | --- | --- |
| **Health outcomes** | | **Health determinants** | | | |
| **Perceived community health issues** | **Morbidity** | **Health care (access & quality)** | **Health behaviors** | **Demographics & social environment** | **Physical**  **environment** |
| Major community health problems [41, 52, 53, 55, 57, 89, 93, 94, 96, 104, 106, 121] | Overall health status [28, 34, 35, 43, 64, 65, 67, 71, 74, 93, 129, 136] | Health insurance coverage [19, 52, 57, 64, 66, 77, 106, 129, 161] | Tobacco use/ Smoking [19, 51, 52, 57, 64, 65, 71, 77, 93, 116, 129, 159] | Age | Air quality |
| Health/Social conditions that needed to be better addressed [21, 28, 41, 53, 55, 57, 94] | High blood pressure [28, 43, 48, 52, 57, 65, 66, 71, 76, 77, 116] | Health seeking behavior [8, 21, 35, 53, 57, 64, 67, 71, 76, 94, 96, 104, 116, 123, 161] | Physical activity [19, 28, 52, 53, 57, 64, 65, 136, 157] | Sex | Water quality [28, 75, 116] |
| Perceptions of community safety [51, 55, 74, 121] | Hyperlipidemia [52, 65, 66, 136] | Utilization of hospital services [8,21, 28, 34, 53, 64, 71, 77, 93, 96, 106] | Eating behaviors [19, 28, 43, 52, 53, 57, 66, 76, 93, 157] | Race/Ethnicity | Housing [75, 116] |
|  | Hepatitis B [28, 57, 65] | Utilization of outpatient services [8, 21, 34, 53, 57, 64, 67, 71, 77, 94, 96, 116] | Sexual behaviors [47, 116] | Income | Access to toilet or latrine facilities [28, 75] |
|  | Cancer [52, 57, 66] | Waiting time for outpatient visits [57, 71] | Alcohol use [19, 47, 51, 52, 57, 64, 66, 77, 93, 116] | Poverty level | Garbage [75] |
|  | Diabetes and metabolic disorders [19, 28, 43, 66, 71, 76, 77, 159] | Language barrier [57, 77] | Anthropometry [40, 93, 116] | Educational attainment | Access to electricity [116] |
|  | Sexual transmitted diseases (STDs) [19, 48, 76, 123] | Interest in using selected services [71, 106] | Immunizations, check-up, clinical test, vaccination, and screenings [19, 28, 43, 47, 53, 57, 64, 65, 75-77, 116] | Employment status | Traffic, crime and violence [53, 161] |
|  | AIDS/HIV [19, 48, 116] | Satisfaction with health services [8, 21, 28, 64, 93, 116, 129] | Food insecurity [57, 93] | Foreign born | Transportation [28, 53] |
|  | Obesity and overweight [48, 65, 66, 76, 93, 116, 136] | Barriers faced receiving health services [21, 34, 35, 43, 48, 53, 66, 67, 71, 77, 93, 94, 129] | Need for health information [89, 94] | Access to telephone, dependable transportation, internet |  |
|  | Tuberculosis [28, 116] | Appropriateness of health and social services [21, 41] | Seat belt use [161] | Homelessness |  |
|  | Heart diseases and stroke  [19, 52, 57, 71, 89, 159] | Using alternative health methods [64] |  | Language spoken at home |  |
|  | Infectious diseases [75, 76] |  |  | Marital status |  |
|  | Asthma [52, 57, 66, 89, 136] |  |  | Number of children in home |  |
|  | Epilepsy [116] |  |  | Height/Weight |  |
|  | Depression and mental health illness [43, 48, 57, 66, 71, 76, 77, 89, 93] |  |  | Connection to community [28, 74] |  |
|  | Arthritis or osteoporosis [52, 57, 66, 76, 116] |  |  | Community participation/Civic engagement [28, 51, 74, 89, 104] |  |
|  | Kidney diseases [57, 76] |  |  | Social capital/Social Support [74, 76, 77, 89] |  |
|  | Back pain/Musculoskeletal diseases [71, 89] |  |  |  |  |
|  | Headaches/Migraines [71, 89] |  |  |  |  |
|  | Exposure to violence [116] |  |  |  |  |
|  | Anemia [28, 76] |  |  |  |  |
|  | Allergies [66] |  |  |  |  |
|  | Pregnancy complications [76] |  |  |  |  |
|  | Disability [28, 123] |  |  |  |  |
|  | Substance use [48] |  |  |  |  |
| *Numbers in parenthesis indicate the number of references | | | | | |
